# Clinical and Radiological Characteristics of Pediatric Skull Fractures and Their Association with Neurosurgical Intervention: A Retrospective Cohort Study

**DOI:** 10.3390/children13091264

**Published:** 2026-09-17

**Authors:** Merve Ağaçkıran, Baylar Baylarov, Murat Gölpınar, Mustafa Cemil Kılınç, İlter Ağaçkıran

**Affiliations:** 1 Department of Emergency Medicine, Çorum Erol Olçok Training and Research Hospital, Çorum 19200, Turkey; 2 Department of Neurosurgery, Çorum Erol Olçok Training and Research Hospital, Çorum 19200, Turkey; 3 Department of Anatomy, Hitit University Faculty of Medicine, Çorum 19030, Turkey; 4 Department of Neurosurgery, Hitit University Faculty of Medicine, Çorum 19030, Turkey; 5 Department of Emergency Medicine, Hitit University Faculty of Medicine, Çorum 19030, Turkey

**Keywords:** pediatric skull fractures, traumatic brain injury, neurosurgical intervention, pneumocephalus, emergency department

## Abstract

**Highlights:**

**What are the main findings?**

- Only 9% of children with CT-confirmed skull fractures required neurosurgical intervention, while most were managed without surgery.
- Older age, intracranial hemorrhage, and depressed skull fracture retained significant associations with neurosurgical intervention in the reduced Firth regression model.

**What are the implications of the main findings?**

- Neurosurgical risk assessment should integrate intracranial injury, fracture morphology, and neurological status rather than rely on an isolated radiological finding.
- These findings may help identify children requiring closer neurosurgical evaluation, but larger multicenter studies are needed before they can be used for clinical prediction or decision thresholds.

**Abstract:**

**Background:** Traumatic brain injury is a major cause of morbidity in children. This study evaluates pediatric patients with skull fractures in the emergency department to determine independent factors associated with neurosurgical intervention. **Methods:** This single-center retrospective study included 133 patients under 18 years old with CT-confirmed skull fractures following head trauma. Demographics, trauma mechanisms, neurological status, fracture characteristics, and intracranial injuries were recorded. Firth penalized logistic regression examined factors associated with surgery. **Results:** The mean age was 4.47 years, and falls were the most common trauma mechanism (63.9%). Pneumocephalus and intracranial hemorrhage were each present in 24.8% of patients. In total, 12 patients (9.0%) required neurosurgical intervention. In univariate analyses, depressed fractures, comminuted fractures, pneumocephalus, and intracranial hemorrhage were strongly associated with intervention. In the multivariable model, age, intracranial hemorrhage and depressed fracture were the factors retaining statistical significance (*p* = 0.017, *p* = 0.022, *p* = 0.013). **Conclusions:** Intracranial hemorrhage showed the strongest independent statistical association with neurosurgical intervention. Although depressed, comminuted fractures and pneumocephalus were associated with surgery in univariate analyses, their independent contributions could not be reliably established due to limited surgical events. Clinical decisions must integrate neurological status and fracture characteristics.

## 1. Introduction

Traumatic brain injury (TBI) is one of the leading causes of morbidity and mortality in the pediatric population and represents a major public health problem affecting more than 3 million children worldwide annually [1]. Mild traumatic brain injury (mTBI), which constitutes the vast majority of pediatric head traumas, accounts for approximately 70–90% of all cases [1]. Although classified as “mild,” approximately 7.5% of these patients have intracranial pathologies detectable by computed tomography (CT), a condition defined as “complicated mTBI” [2,3,4,5,6]. Among the most common pathological findings detected on CT in pediatric mTBI cases are skull fractures, epidural hematoma (EDH), subdural hematoma (SDH), traumatic subarachnoid hemorrhage (tSAH), pneumocephalus, cerebral contusion, and brain edema [5,6]. In particular, certain radiological findings such as pneumocephalus may be associated with skull base fractures or sinus injuries and should be carefully evaluated for infection risk and the need for surgical intervention [7,8,9,10].

Skull fractures are among the most frequently encountered radiological findings in pediatric head traumas and present a broad clinical spectrum. Due to the flexibility of the skull bones and the unclosed sutures in children, the biomechanical properties of these fractures differ from those of adults [11,12]. Most isolated skull fractures follow a benign course, and discharge may be possible after a short period of observation [13,14]. However, in some cases, these fractures may be accompanied by intracranial hemorrhages, contusions, or pneumocephalus, which makes the clinical course more unpredictable and negatively affects the prognosis [5,6,15,16]. On the other hand, it is known that the use of CT in pediatric patients is associated with a long-term risk of malignancy due to ionizing radiation exposure [17,18,19]. Therefore, accurately determining which patients require imaging and the early recognition of high-risk patients are of paramount importance. Although there are studies in the literature regarding the management of isolated skull fractures, studies evaluating the independent effects of the radiological characteristics of skull fractures (fracture type, localization) and accompanying intracranial findings (hemorrhage, pneumocephalus) on surgical requirement using advanced statistical models are limited. The aim of this study is to evaluate the demographic, clinical, and radiological characteristics of pediatric skull fracture cases presenting to the emergency department and to determine the factors associated with the need for neurosurgical intervention. Specifically, the relationship between fracture morphology, pneumocephalus, intracranial hemorrhage, and surgical requirement was investigated.

## 2. Materials and Methods

### 2.1. Study Design

This study was designed as a single-center retrospective observational study. The study was conducted in the Emergency Department of Hitit University Faculty of Medicine Çorum Erol Olçok Training and Research Hospital. Patients under 18 years of age who presented to the emergency department due to head trauma between 1 January 2022 and 30 September 2025 and were found to have a skull fracture via computed tomography (CT) were included in the study. Patients with previously known neurological diseases or a history of cranial surgery, those with incomplete clinical or radiological data, and multisystem trauma patients requiring emergency surgical intervention for reasons other than head trauma were excluded from the study.

Patient demographics, post-traumatic seizures, and admission neurological status—evaluated using the age-appropriate Pediatric Glasgow Coma Scale (PGCS) [3] and the presence of focal neurological deficits—were retrospectively collected from the hospital’s electronic database. Cranial computed tomography (CT) scans were evaluated to determine fracture localization, side, and pattern (linear vs. comminuted) [20,21]. Skull fractures were morphologically classified as non-depressed or depressed. Depressed skull fractures (DSFs) were further categorized using Choux’s radiological classification into true, flat, or ping-pong ball fractures [22,23], with the maximum depth of bone depression measured in millimeters (mm) on axial CT scans [23]. Associated intracranial pathologies (epidural (EDH) or subdural hematoma (SDH), traumatic subarachnoid (tSAH) or intracerebral hemorrhage (ICH), cerebral contusion, and pneumocephalus) were documented [21]. Fractures were defined as ‘complicated’ if accompanied by at least one associated intracranial injury/pathology, and ‘isolated’ if no accompanying pathology was present on CT [20]. Neurosurgical intervention was defined as any operative procedure performed due to the skull fracture or associated intracranial injuries [21,23]. Standard institutional criteria for surgery included: (1) significant mass effect (midline shift, basal cistern compression) caused by hemorrhage, (2) progressive neurological deterioration, (3) open/compound depressed fractures carrying infection risk, or (4) depressed fractures where the depth of depression exceeded the thickness of the adjacent intact skull or caused dural disruption [22,23]. The analysis included acute neurosurgical procedures following the index emergency department presentation and hospitalization, excluding subsequent elective procedures for residual cosmetic deformities. Surgical indications could overlap, and a single primary indication could not consistently be identified from retrospective records. No patient underwent invasive intracranial pressure (ICP) monitoring because of institutional infrastructure and equipment limitations. Without direct ICP measurements, possible pressure-related indications were assessed indirectly from documented clinical and radiological findings, including neurological status, intracranial hematoma, and mass effect.

In accordance with national regulations, all patients included in this study were registered as medico-legal cases and reported to the relevant judicial authorities. The available medical and medico-legal records were also reviewed to determine whether any child protection measures had been implemented.

The primary endpoint of the study is the need for neurosurgical intervention. Secondary endpoints are hospitalization and the presence of intracranial complications such as pneumocephalus and intracranial hemorrhage.

### 2.2. Statistical Analysis

The conformity of the data to a normal distribution was examined using the Kolmogorov–Smirnov test when *n* ≥ 50 according to the groups, and using the Shapiro–Wilk test when *n* < 50. The Mann–Whitney U test was used to compare non-normally distributed data between groups. Fisher’s exact test was utilized in comparing categorical data between groups if any of the expected observation values was less than 5. The Pearson chi-square test was used to compare categorical data in the case of multiple responses. Multiple comparisons of proportions were examined with the Bonferroni-corrected Z-test and presented using a lettering method.

In the logistic regression analysis, Firth’s penalized maximum-likelihood estimation was used to reduce the risk of bias in maximum-likelihood estimates, the possibility of quasi-complete separation, and the impact of a small sample size. This method reduces the bias seen in parameter and variance estimates and improves model convergence. Given the limited number of neurosurgical events (*n* = 12), the multivariable analysis was considered exploratory and was intended to assess adjusted associations rather than to develop or validate a clinical prediction model.

The analysis results were presented as mean ± standard deviation, median (minimum–maximum) for quantitative data, and as frequency (percentage) for categorical data. A significance level of “*p* < 0.05” was considered in all calculations. IBM SPSS 26 (IBM Corp., Armonk, NY, USA, Released 2019) software and R software (Version 4.5.1, R Core Team, Vienna, Austria, 2025) were used to obtain the analysis findings. In the R software, the findings were obtained using the logistf package [24].

## 3. Results

**Figure 1:**
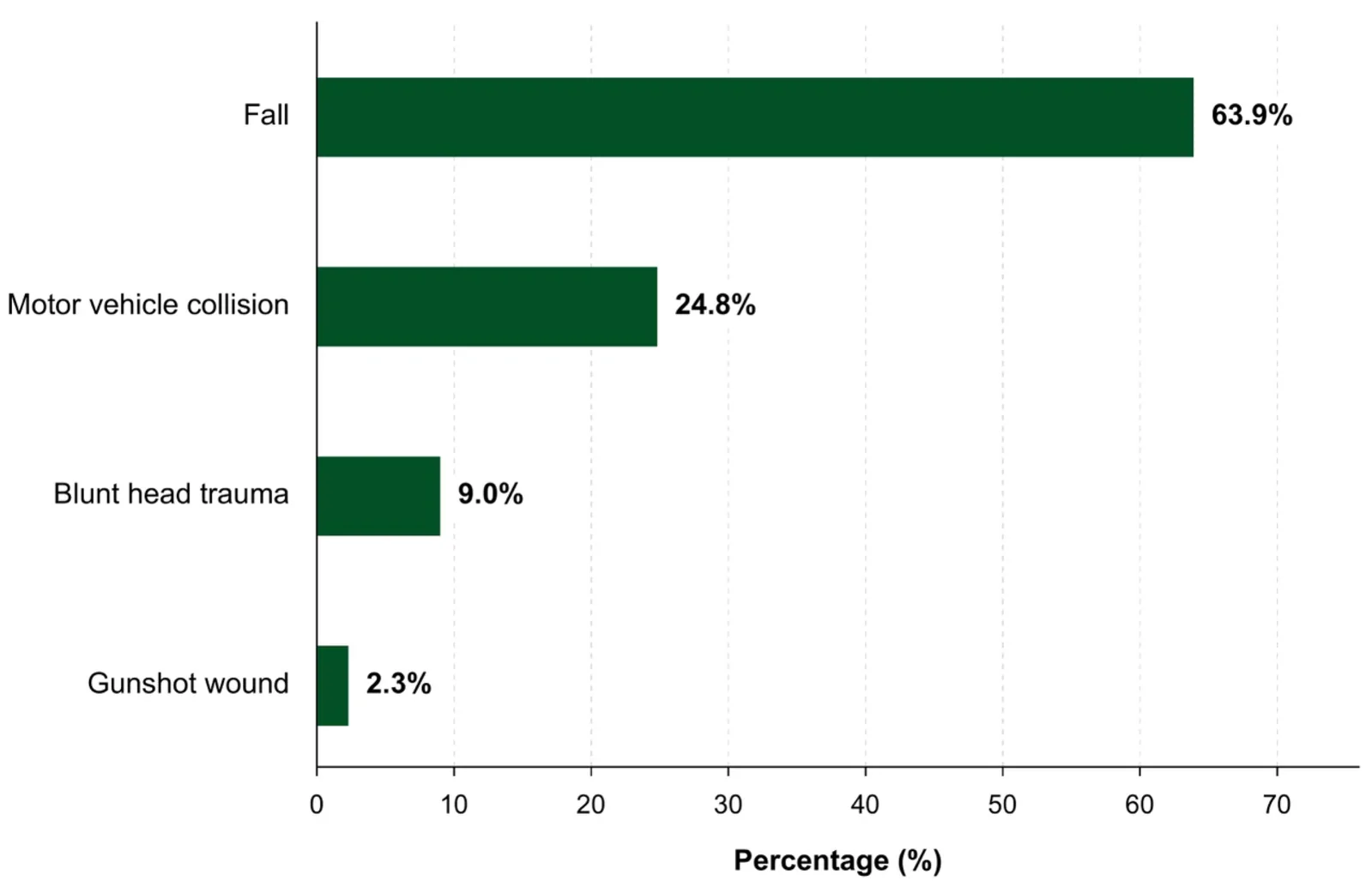
Distribution of injury mechanisms in the study cohort.

The mean age of the 133 patients included in the study was 4.47 years (age range: 0–17 years). Female children constituted 42.1% of the patients, while male children accounted for 57.9%. Regarding trauma mechanisms, the vast majority of patients presented due to falls (63.9%), followed by traffic accidents (24.8%), direct impact (9.0%), and gunshot wounds (2.3%) (Figure 1). Among the 133 patients, 119 (89.47%) had a PGCS score of 15, 8 (6.02%) had scores between 9 and 14, and 6 (4.51%) had scores of 8 or lower. Focal neurological deficits were observed in 3 patients (2.25%), including left hemiparesis in 2 (1.50%) and right third cranial nerve palsy in 1 (0.75%), while loss of consciousness was documented in 4 patients (3.01%). In the subsequent clinical and radiological evaluations, pneumocephalus and intracranial hemorrhage were both detected in 24.8% of the patients (associated intracranial injuries were identified in 33 patients (24.81%), including EDH in 14 (10.53%), SAH in 12 (9.02%), cerebral contusion in 10 (7.52%), ICH in 4 (3.01%), SDH in 3 (2.26%), and IVH in 3 (2.26%); some patients had multiple concomitant injuries). The incidence of post-traumatic seizures was extremely low, observed in only one patient (0.8%). Regarding clinical outcomes, the overall hospitalization rate was determined to be 93.2%, and 12 patients (9.0%) required neurosurgical intervention (Table 1). No in-hospital mortality was observed among the 133 patients included in the study.

**Figure 2:**
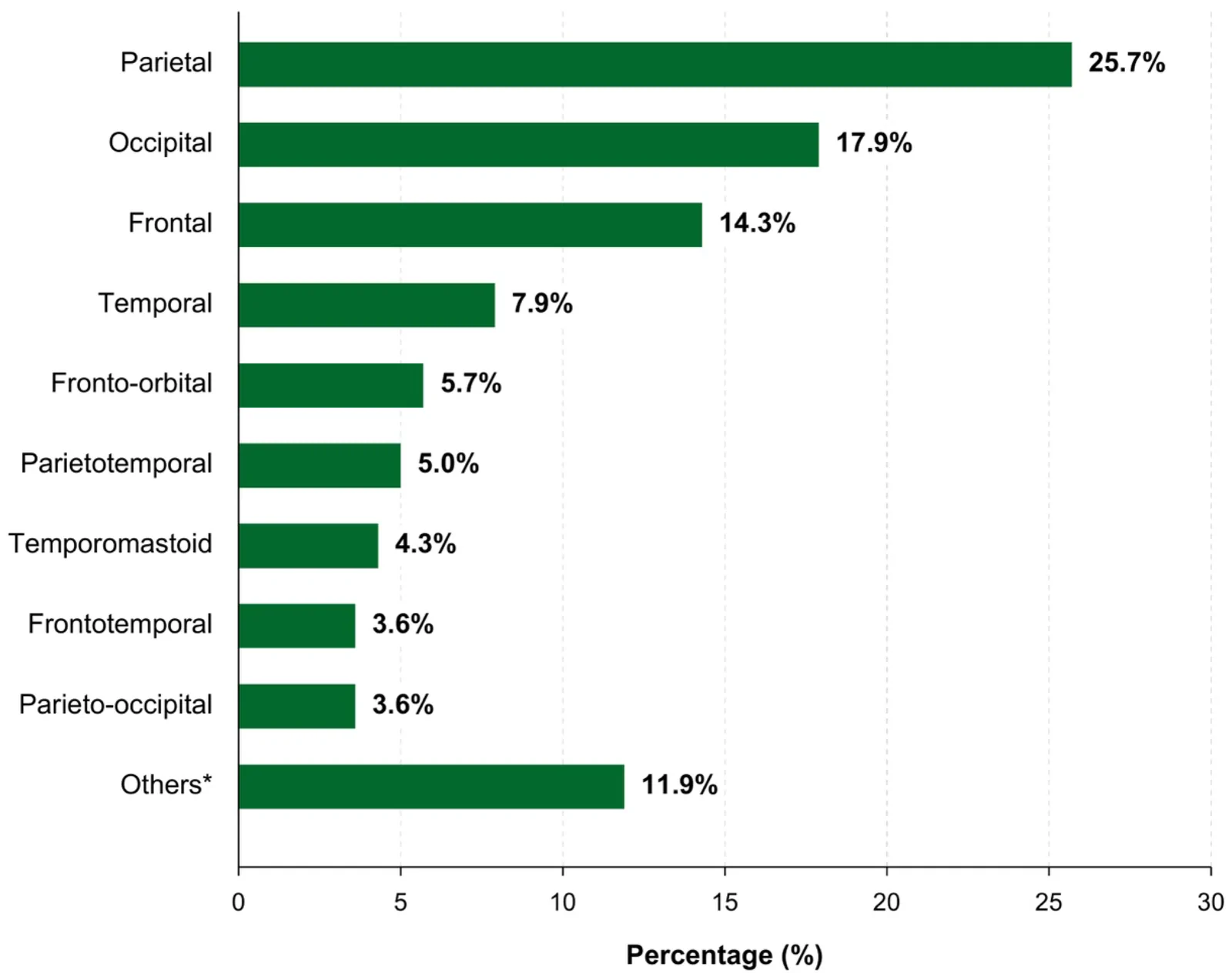
Distribution of anatomical locations of skull fractures. *: The distribution of fractures grouped under “Others” is presented in detail in Table 2.

It was observed that 91.7% of the patients had a single fracture, whereas multiple fractures were detected in only 8.3%. Non-depressed fractures were present in 76.7% of the patients, and DSF in 23.3%. Within the subgroup of 31 patients with DSF, 19 (61.29%) presented with simple fractures and 12 (38.71%) had compound fractures. Radiological subclassification of these depressed cases revealed true depressed fractures in 18 patients (58.06%), flat depressed fractures in 12 (38.71%), and a ping-pong ball fracture in 1 (3.23%). Linear fractures were observed in 80.5% of the patients, while comminuted fractures were observed at a rate of 19.5%. When examining the distribution of fracture localizations, parietal fractures were found to be the most common with a rate of 25.7%. This was followed by occipital (17.9%) and frontal (14.3%) fractures. Less frequently, these were followed by temporal (7.9%), fronto-orbital (5.7%), temporoparietal (5%), and other mixed-distribution localizations (Figure 2). When examining the side distributions, it was observed that 56% of the fractures were on the right side and 34.3% on the left side, 6% were bilateral, and 3.7% had a midline location (Table 2).

There is a statistically significant difference between the ages of the patients regarding the need for surgery (*p* < 0.001). While the median age value for patients who did not need surgery was 2 years, the median age value for patients who needed surgery was 8 years. There is no statistically significant difference between gender distributions according to the presence of surgery (*p* = 0.559). While the pneumocephalus rate in patients who did not require surgery was 21.5%, the pneumocephalus rate in patients requiring surgery was 58.3% (*p* = 0.010). While the hemorrhage rate in patients who did not require surgery was 20.7%, the hemorrhage rate in patients requiring surgery was 66.7% (*p* = 0.002). A detailed analysis of patients requiring surgery is given in Table 3.

**Figure 3:**
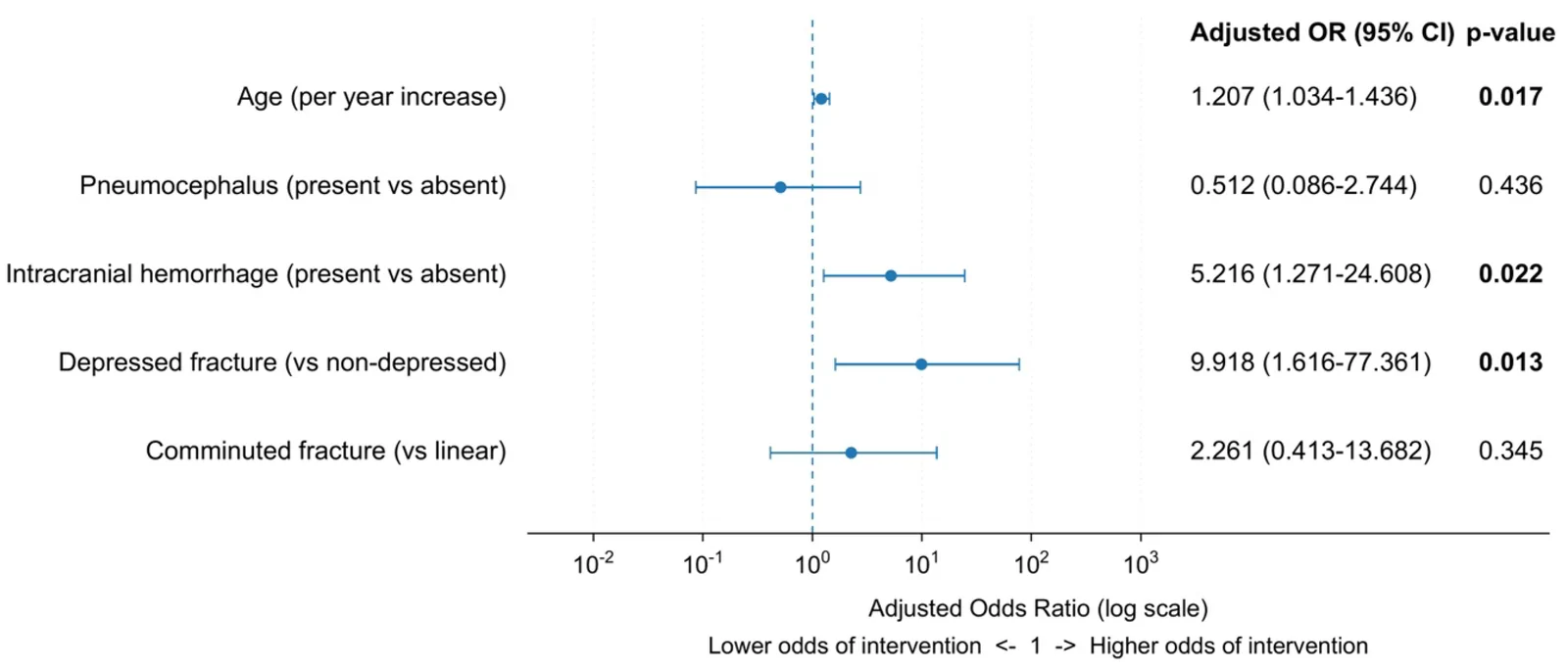
Forest plot of multivariable Firth penalized logistic regression analysis showing adjusted odds ratios (ORs) and 95% confidence intervals for factors associated with neurosurgical intervention. (Model 1).

**Figure 4:**
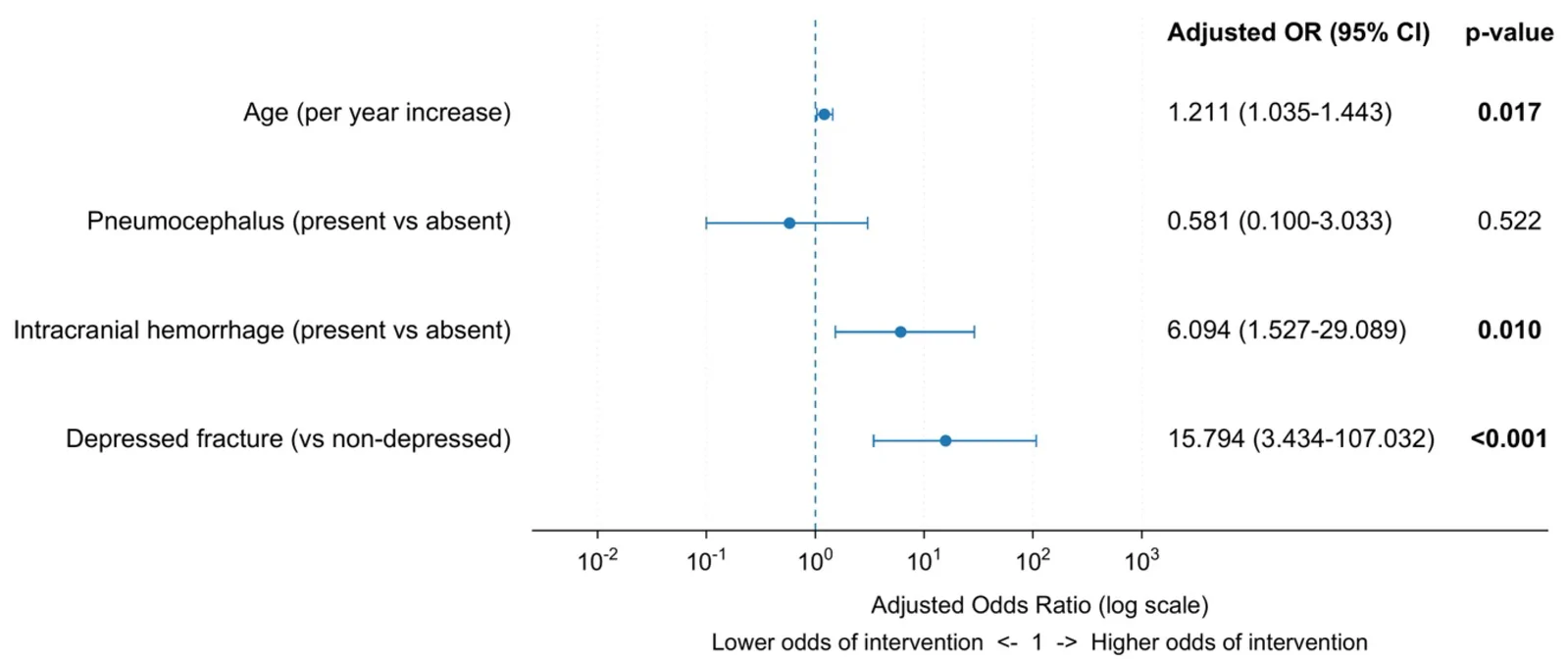
Forest plot of multivariable Firth penalized logistic regression analysis showing adjusted odds ratios (ORs) and 95% confidence intervals for factors associated with neurosurgical intervention. (Model 2).

In Table 4, the effects of the factors found to be associated with the presence of surgery were investigated. Firth’s penalized maximum-likelihood estimation was used in the logistic regression analysis to mitigate the small sample size (number of patients undergoing surgery *n* = 12), the probability of quasi-complete separation, and the risk of bias in maximum-likelihood estimates. Due to the structural characteristics of this method, standard errors relatively increase and confidence intervals can widen. This situation is an expected methodological outcome, reducing the risk of biased estimation and allowing for more reliable inferences to be made. As a result of the univariate analysis, as the age of the patients increases, the likelihood of surgery increases significantly (OR = 1.202; 95% CI: 1.079–1.353; *p* = 0.001). It was determined that the presence of pneumocephalus (OR = 4.914; 95% CI: 1.518–16.893; *p* = 0.008) and the presence of hemorrhage (OR = 7.148; 95% CI: 2.181–26.584; *p* = 0.001) significantly increased the likelihood of surgery. It was observed that a depressed fracture (OR = 18.387; 95% CI: 4.897–100.522; *p* < 0.001) and a comminuted fracture (OR = 16.053; 95% CI: 4.585–69.726; *p* < 0.001) significantly increased the likelihood of surgery. The overall fit of the multivariate model created with the factors found significant as a result of the univariate analysis was found to be statistically significant (Chi-square = 35.070; *p* < 0.001). As a result of the multivariate analysis, it was seen that the age factor is independently borderline insignificant (OR = 1.207; 95% CI: 1.034–1.436; *p* = 0.017). The presence of hemorrhage continued to increase the likelihood of surgery by approximately 5 times even when controlled with other variables (OR = 5.216; 95% CI: 1.271–24.608; *p* = 0.022). Table 5 presents the optimized and reduced Firth penalized logistic regression model, taking into account the high uncertainty and intervariate relationships observed in the full model created in Table 4. Due to the very wide confidence interval for the fracture pattern variable in the full model and its high correlation with fracture morphology, the fracture pattern variable was removed from Model 2 to reduce multicollinearity and uncertainty in model predictions. Thus, age, pneumocephalus, intracranial hemorrhage, and fracture morphology variables were retained in the model. The forest plot of the table is provided in Figure 3 and Figure 4.

A detailed analysis of the surgical indications and procedures for the 12 operated patients is provided in Table 6. The most frequently documented indications were open/compound depressed fracture for infection prevention and depth of depression and/or dural disruption (9 patients [75.0%] each). Underlying brain parenchymal injury and/or neurological status was recorded as an indication in 3 patients (25.0%), and intracranial hematoma and/or mass effect in 4 (33.3%). Indication categories were not mutually exclusive; 5 patients (41.7%) had at least one of the latter two indications. No cosmetic or late-complication indication was recorded for these acute procedures. Admission PGCS was 15 in 5 patients (41.7%), 9–14 in 4 (33.3%), and ≤8 in 3 (25.0%). Neurological abnormalities were documented in 3 operated patients: loss of consciousness in 2 and left hemiparesis in 1. The most frequently performed neurosurgical procedures included craniotomy (75%), surgical debridement (66.6%), and duraplasty (58.3%). Furthermore, epidural hematoma evacuation was necessary in 50% of the surgical cases. The relationship between the depth of depressed skull fractures and the rate of surgery is detailed in Table 7, demonstrating a sharply increased need for intervention in fractures depressed by more than 1 cm (53.3%).

All 133 patients presenting to the emergency department were registered as medico-legal cases and reported to the relevant judicial authorities in accordance with national regulations. A review of the available records revealed that three patients (2.3%), aged 1, 14, and 15 years, were placed under child protection. Regarding the mechanisms of injury, one patient had sustained a fall at home, while the other two had been involved in motor vehicle collisions. None of these patients underwent surgical intervention. One patient had a displaced skull fracture, whereas the other two had non-displaced skull fractures. Regarding associated intracranial injuries, one patient had a cerebral contusion accompanied by traumatic subarachnoid hemorrhage, another had isolated traumatic subarachnoid hemorrhage, and the third had no hemorrhagic complications. All three patients had a Glasgow Coma Scale score of 15 at presentation; however, one exhibited third cranial nerve palsy.

## 4. Discussion

In the pediatric population, skull fractures are one of the most frequently encountered radiological findings in mTBI cases, and their clinical management varies greatly depending on whether they are isolated or complicated. Biomechanical differences such as the flexibility of the skull bones and open sutures in children directly affect fracture morphology and the energy transmitted to the brain parenchyma. In this study, the radiological characteristics of pediatric skull fractures and the effects of accompanying conditions such as pneumocephalus and intracranial hemorrhage on surgical requirement and clinical prognosis were evaluated.

Against this biomechanical background, we observed a statistically significant age difference between patients who underwent surgery and those managed conservatively, with median ages of 8 and 2 years, respectively (*p* < 0.001). This difference may partly reflect age-dependent changes in pediatric cranial anatomy. In infants and very young children, the greater pliability and elasticity of the cranial bones, together with open sutures and fontanels, facilitate the absorption and distribution of traumatic forces, potentially reducing focal force transmission to the underlying brain parenchyma [21]. Extradural hematomas are also uncommon in children younger than 2 years, accounting for approximately 1.5–3% of cases in large pediatric series, possibly because of the firm adherence of the dura mater to the inner table and suture lines and the absence of well-developed bony grooves surrounding the meningeal arteries [22]. Conversely, the incidence of compound depressed fractures, which more frequently require surgery because of the risks of infection and dural injury, increases with age [23]. Therefore, the predominance of younger children in our non-surgical group may reflect both the protective anatomical characteristics of early childhood and a clinical preference for conservative management when appropriate [21].

Pneumocephalus was observed at a notable frequency in our cohort, and falls were the most common mechanism of trauma, consistent with epidemiological studies identifying falls and motor vehicle collisions (MVCs) as the leading causes of pediatric head injury [3,15]. Pneumocephalus most commonly develops following traumatic skull fractures, particularly skull base fractures [6,25]. Although its incidence was reported as 0.5–1% in earlier studies [6,8], the widespread use of CT has enabled the detection of smaller intracranial air collections and may explain the higher rates reported in contemporary series [26].

In our cohort, univariate analysis showed that depressed skull fractures (OR = 18.387), comminuted fracture patterns (OR = 16.053), and pneumocephalus (OR = 4.914) were significantly associated with neurosurgical intervention. However, in the multivariable Firth penalized logistic regression model, only intracranial hemorrhage retained a statistically significant adjusted association with neurosurgical intervention (OR = 5.420, *p* = 0.018), whereas fracture morphology and pneumocephalus were no longer significant after adjustment. The multivariable loss of statistical significance for fracture morphology should not be interpreted as evidence that depressed or comminuted fractures are clinically unimportant or cannot constitute operative indications. Rather, given the small number of surgical events and the wide confidence intervals, the present model was unable to reliably determine their independent statistical contribution after adjustment. Pneumocephalus is frequently associated with epidural, subdural, and intraparenchymal hemorrhages [18,26], but its clinical significance depends largely on whether it occurs in isolation or with other intracranial lesions [25]. The prognosis of isolated pneumocephalus is generally favorable, and serious complications are uncommon [25]. Blanchard et al. reported 0% mortality and no requirement for neurosurgical intervention among children with isolated traumatic pneumocephalus [25]. Similarly, isolated pediatric skull fractures without associated intracranial injuries rarely result in delayed complications or require surgical treatment [20]. Taken together, these findings indicate that our multivariable model could not reliably determine the independent statistical contribution of fracture morphology, while the clinical significance of pneumocephalus should be interpreted in the context of associated injuries and the overall neurological status [25].

In our cohort, surgery was performed in 53.3% of patients with a fracture depression > 1 cm, compared with 10.0% and 20.0% in the <0.5-cm and 0.5–1.0-cm groups, respectively. However, because fracture depth was itself considered as part of the institutional criteria for surgical intervention, these findings should be interpreted as descriptive of our institutional surgical decision-making rather than as validation of an independent or universally applicable depth threshold. It has been emphasized that deeper bone depressions correlate with a higher risk of both dural tears and cortical lacerations, resulting in a worse prognosis [23]. Dural and brain lacerations are reported in 24.3% of operated pediatric cases, with compound depressed fractures leading to significantly more brain lacerations than simple fractures (29% vs. 15.5%) [23]. However, a conservative, non-surgical approach is strongly advocated in simple (closed) depressed fractures without associated intracranial hematomas and where the bone depression is 1 cm or less [23]. The alignment of our surgical rate (53.3%) for depressions exceeding 1 cm with these established criteria validates our clinical adherence to safe conservative limits for shallow, closed depressions in pediatric patients, thereby avoiding unnecessary craniotomies.

Surgical indications overlapped and most commonly involved open/compound depressed fractures requiring infection prevention or substantial depression and/or dural disruption. Five of the 12 operated patients had indications related to parenchymal injury, neurological status, intracranial hematoma, or mass effect, indicating that associated clinical and radiological findings also contributed to surgical decisions. No cosmetic indication was documented for the acute procedures studied; subsequent elective cosmetic correction was not assessed.

Most traumatic brain injuries are classified as mild (GCS 13–15) [7,17], and 89.47% of our patients presented with a PGCS score of 15; nevertheless, the hospitalization rate was 93.2%. Although neurological deterioration is uncommon in mild head trauma, the risk is not negligible [17], and this high admission rate may reflect concerns regarding delayed intracranial complications and potential non-accidental trauma (NAT) [20]. Accurate risk stratification is therefore essential, and clinical decision rules such as PECARN can reduce unnecessary CT use in low-risk patients [3]. Although CT remains the diagnostic gold standard, childhood radiation exposure has been associated with an increased risk of cancer [10,16], supporting selective imaging based on clinical indications [1,3,4]. Similarly, routine hospitalization is often unnecessary in neurologically stable patients with normal CT findings [2] or isolated skull fractures [27]. Children with isolated fractures, a GCS score of 15, normal neurological findings, adequate oral intake, and no suspicion of NAT may be safely discharged [20]. Evidence-based protocols can reduce admissions by approximately 25% without compromising safety [28]. Although previous studies suggest that selected neurologically stable children with isolated skull fractures may be safely discharged after appropriate observation, the present study was not designed to evaluate discharge strategies or compare outcomes between admitted and discharged patients.

Pediatric skull fractures, particularly in infants and very young children, necessitate consideration of both non-accidental injury (NAI) and abuse-related head trauma (AHT). Certain findings, such as subdural hematoma, complex or multiple skull fractures, associated intracranial injuries, and a history inconsistent with the child’s developmental stage, should raise clinical concern for possible abuse. Three patients (2.3%) were placed under child protection, although all had an admission Glasgow Coma Scale score of 15 and none underwent surgical intervention. These observations highlight the importance of considering safeguarding needs beyond the level of consciousness or the need for neurosurgical treatment. Therefore, radiological findings should not be interpreted in isolation, and the mechanism of injury, the child’s developmental abilities, physical examination findings, and social history should be considered together when evaluating the possibility of NAI. Emergency department assessment should also address the child’s living environment, caregiving circumstances, and potential safety concerns through sensitive, age-appropriate history-taking. In cases of suspected abuse, multidisciplinary evaluation and referral to child protection services are necessary. The recorded injury mechanisms in these cases were a fall at home and motor vehicle collisions; however, child protection measures alone do not establish non-accidental injury, and these cases should not be interpreted as confirmed abusive head trauma.

Some important limitations should be considered when interpreting our findings. First, the single-center retrospective design limits generalizability, as CT utilization, hospitalization policies, and neurosurgical indications may vary across institutions. Second, the limited sample size (*n* = 133), particularly the small number of surgical events (*n* = 12), restricted subgroup and multivariable analyses. Although Firth penalized logistic regression was used to reduce small-sample bias and address quasi-complete separation, it cannot eliminate the substantial imprecision resulting from only 12 surgical events. Accordingly, the multivariable analysis should be considered exploratory, as reflected by the wide confidence intervals of several adjusted estimates. Third, retrospective data collection may be subject to information bias because minor clinical symptoms and trauma characteristics may have been incompletely documented; similarly, clinical decision rules such as PECARN could not be prospectively assessed. In addition, indication bias should be considered because several radiological variables evaluated as factors associated with surgery—including open/compound depressed fractures, fracture depth, dural disruption, and hematoma or mass effect—were themselves components of institutional surgical decision-making. Accordingly, these associations should be interpreted as reflecting factors associated with institutional neurosurgical management rather than independently established surgical indications. Finally, long-term neurocognitive, psychomotor, and developmental outcomes were not available, and prospective multicenter studies with longer follow-up are needed to confirm these findings. The restriction to acute procedures limits conclusions about later cosmetic surgery. Retrospective records did not consistently identify a primary surgical indication or permit reliable quantification of neurological deterioration or the contributions of surgeon preference and family discussions. Invasive intracranial pressure (ICP) monitoring was unavailable because of institutional infrastructure and equipment limitations. Consequently, pressure-related indications could not be verified by direct measurements and could only be assessed indirectly from documented clinical and radiological findings.

## 5. Conclusions

Most pediatric skull fractures in our cohort were managed without neurosurgical intervention. Intracranial hemorrhage was the only factor that retained a statistically significant association with neurosurgical intervention in the multivariable Firth model. Depressed and comminuted fractures and pneumocephalus were associated with intervention in univariate analyses, but their independent statistical contributions could not be reliably established after adjustment. Because only 12 surgical events occurred and the adjusted estimates had wide confidence intervals, these findings should be considered exploratory and require validation in larger multicenter cohorts. Clinical decisions should therefore integrate neurological status, fracture morphology and depth, dural integrity, and associated intracranial injuries rather than rely on any single statistical association.

## Figures and Tables

**Table 1:** Baseline demographic and clinical characteristics of the study cohort.

Characteristic	Value
**Age (years)**	
Mean ± SD	4.47 ± 4.76
Median (range)	2 (0–17)
**Sex, *n* (%)**	
Female	56 (42.1)
Male	77 (57.9)
**Mechanism of injury, *n* (%)**	
Fall	85 (63.9)
Motor vehicle collision	33 (24.8)
Blunt head trauma	12 (9.0)
Gunshot injury	3 (2.3)
**Clinical findings, *n* (%)**	
Pneumocephalus	33 (24.8)
Intracranial hemorrhage	33 (24.8)
Seizure	1 (0.8)
**Clinical outcomes, *n* (%)**	
Neurosurgical intervention	12 (9.0)
Hospitalization	124 (93.2)

Data are presented as mean ± standard deviation, median (range), or *n* (%).

**Table 2:** Fracture characteristics of the study cohort.

	*n*	%
**Number of fractures**		
Single	122	91.7
Multiple	11	8.3
**Fracture morphology**		
Non-depressed	102	76.7
Depressed	31	23.3
**Fracture pattern**		
Linear fracture	107	80.5
Comminuted fracture	26	19.5
Fracture localization *		
Anterior skull base	2	1.4
Frontal	20	14.3
Fronto-orbital	8	5.7
Fronto-orbito-ethmoidal	2	1.4
Fronto-orbito-nasal	1	0.7
Frontotemporal	5	3.6
Fronto-temporo-parietal	3	2.1
Clivus	1	0.7
Occipital	25	17.9
Occipito-temporo-mastoid	2	1.4
Parietal	36	25.7
Parieto-occipital	5	3.6
Parietotemporal	7	5.0
Parieto-temporo-occipital	1	0.7
Sphenoid sinus	1	0.7
Temporal	11	7.9
Temporomastoid	6	4.3
Temporo-occipital	2	1.4
Temporoparietal	2	1.4
Fracture side *		
Right	75	56.0
Left	46	34.3
Bilateral	8	6.0
Midline	5	3.7

*: Patients with fractures involving multiple anatomical regions were counted in each relevant category.

**Table 3:** Comparison of Clinical and Radiological Characteristics According to Neurosurgical Intervention.

Variable	No Neurosurgical Intervention (*n* = 121)	Surgery (*n* = 12)	*p*
**Age—Median (min–max)**	2 (0–17)	8 (4–17)	**<0.001 ^m^**
**Sex**			
Female	50 (41.3)	6 (50)	0.559 ^f^
Male	71 (58.7)	6 (50)	
**Mechanism of injury**			
Gunshot	2 (1.7) ^a^	1 (8.3) ^a^	**0.002 ^f^**
Blunt head trauma	10 (8.3) ^a^	2 (16.7) ^a^	
Fall	83 (68.6) ^a^	2 (16.7) ^b^	
Motor vehicle collision	26 (21.5) ^a^	7 (58.3) ^b^	
**Pneumocephalus**	26 (21.5)	7 (58.3)	**0.010 ^f^**
**Hemorrhage**	25 (20.7)	8 (66.7)	**0.002 ^f^**
**Hospitalization**	112 (92.6)	12 (100.0)	1.000 ^f^
**Number of fractures**			
Single	110 (90.9)	12 (100)	0.598 ^f^
Multiple	11 (9.1)	0 (0)	
**Fracture morphology**			
Non-depressed	100 (82.6)	2 (16.7)	**<0.001 ^f^**
Depressed	21 (17.4)	10 (83.3)	
**Fracture pattern**			
Linear	104 (85.9)	3 (25)	**<0.001 ^f^**
Comminuted	17 (14.1)	9 (75)	
Fracture side *			
Right	66 (55.0)	9 (75)	
Left	43 (35.8)	3 (25)	0.462 ^x^
Bilateral	8 (6.7)	0 (0)	
Midline	5 (4.1)	0 (0)	
**Cerebral contusion**	7 (5.8)	3 (25)	**0.047 ^f^**
**Epidural hematoma**	10 (8.3)	4 (33.3)	**0.024 ^f^**
**Subdural hematoma**	2 (1.7)	1 (8.3)	0.249 ^f^
**Subarachnoid hemorrhage**	10 (8.3)	2 (16.7)	0.295 ^f^
**Intraventricular hemorrhage**	2 (1.7)	1 (8.3)	0.249 ^f^
**Intracerebral hemorrhage**	2 (1.7)	2 (16.7)	**0.041 ^f^**
**Documented PGCS other than 15**	8 (6.6)	6 (50.0)	**<0.001 ^f^**
Recorded neurological abnormality **	4 (3.3)	3 (25.0)	**0.016 ^f^**

m: Mann–Whitney U test, Median (min.-max.); x: Pearson chi-square test, f: Fisher’s exact test, *n* (%). a,b: No difference between groups with the same letter (Z test with Bonferroni correction); PGCS, Pediatric Glasgow Coma Scale; *: Some patients had fractures involving more than one anatomical region; **: Recorded neurological abnormality included focal neurological deficits and documented loss of consciousness.

**Table 4:** Univariate and Multivariable Firth Penalized Logistic Regression Analysis for Factors Associated with Neurosurgical Intervention (Model 1—Full Model).

Variable	Univariate OR (95% CI)	*p*	Multivariable OR (95% CI)	*p*
**Age (per year increase)**	1.202 (1.079–1.353)	**0.001**	1.207 (1.034–1.436)	**0.017**
**Mechanism of injury**				
Gunshot	Reference	—	—	—
Blunt head trauma	0.397 (0.032–5.746)	0.467	—	—
Fall	0.050 (0.004–0.684)	**0.028**	—	—
Motor vehicle collision	0.472 (0.054–5.728)	0.512	—	—
**Pneumocephalus**	4.914 (1.518–16.893)	**0.008**	0.512 (0.086–2.744)	0.436
**Intracranial hemorrhage**	7.148 (2.181–26.584)	**0.001**	5.216 (1.271–24.608)	**0.022**
**Fracture morphology**				
Non-depressed	Reference	—	Reference	—
Depressed	18.387 (4.897–100.522)	**<0.001**	9.918 (1.616–77.361)	**0.013**
**Fracture pattern**				
Linear	Reference	—	Reference	—
Comminuted	16.053 (4.585–69.726)	**<0.001**	2.261 (0.413–13.682)	0.345

Abbreviations: OR, odds ratio; CI, confidence interval. Model statistics: likelihood ratio χ^2^ = 35.070, *p* < 0.001.

**Table 5:** Optimized Multivariable Firth Penalized Logistic Regression Model for Predictors of Neurosurgical Intervention (Model 2—Reduced Model).

Variable	Multivariable OR (95% CI)	*p*
**Age (per year increase)**	1.211 (1.035–1.443)	**0.017**
**Pneumocephalus**	0.581 (0.100–3.033)	0.522
**Intracranial hemorrhage**	6.094 (1.527–29.087)	**0.010**
**Fracture morphology**		
Non-depressed	Reference	—
Depressed	15.794 (3.434–107.032)	**<0.001**

Abbreviations: OR, odds ratio; CI, confidence interval. Model statistics: likelihood ratio χ^2^ = 33.897, *p* < 0.001.

**Table 6:** Surgical indications and surgical procedures performed on patients.

	*n*	%
Surgical indication *		
Open/compound depressed fracture and prevention of infection	9	75.0
Depth of depression/disruption of dural integrity	9	75.0
Underlying brain injury/neurological status	3	25.0
Associated intracranial hematoma/mass effect	4	33.3
Surgical Procedure *		
Craniotomy	9	75.0
Decompressive craniectomy	2	16.7
Epidural hematoma evacuation	6	50.0
Surgical debridement	8	66.6
Intracerebral hematoma evacuation	3	25.0
Duraplasty	7	58.3
Cranioplasty	9	75.0
Subdural hematoma evacuation	-	-

* More than one indication/procedure could be recorded for each patient; therefore, percentages may exceed 100%.

**Table 7:** Surgical rates for depressed skull fractures according to their depth.

Depth	No Neurosurgical Intervention (*n* = 20) (%)	Surgery *n* = 10 (%)
<0.5 cm	9 (90.0)	1 (10.0)
0.5–1.0 cm	4 (80.0)	1 (20.0)
>1 cm	7 (46.7)	8 (53.3)

One patient with a ping-pong ball depressed fracture did not have a measurable depression depth and was excluded from this table, bringing the total to 30.

## Data Availability

The data presented in this study can be obtained from the corresponding author upon request, subject to the ethical guidelines of the institution where the study was conducted.
